# Photonic topological semimetals in bianisotropic metamaterials

**DOI:** 10.1038/s41598-019-54523-1

**Published:** 2019-12-04

**Authors:** You-Zhong Yu, Chih-Yu Kuo, Ruey-Lin Chern, C. T. Chan

**Affiliations:** 1 Institute of Applied Mechanics, National Taiwan University, Taipei, 106 Taiwan; 2 Research Center for Applied Sciences, Academia Sinica, Taipei, 115 Taiwan; 3 Department of Physics, Hong Kong University of Science and Technology, Hong Kong, China

**Keywords:** Metamaterials, Nanophotonics and plasmonics, Topological matter

## Abstract

We analyze the photonic topological phases in bianisotropic metamaterials characterized by a chirality tensor with zero trace. We found that the strength of chirality component determines the topological character of the metamaterial. The underlying medium can be considered as a topological semimetal with the nontrivial band gap in the momentum space. The topological properties are described by the spin-orbit Hamiltonians with spin 1 and characterized by the nonzero topological invariants. In particular, photonic quantum Hall states exist when the longitudinal chirality component exceeds the permittivity, whereas photonic quantum spin Hall states are present when the chiral nihility occurs. Considering the dispersion in the frequency domain, the bianisotropic metamaterial is regarded as a photonic Weyl system that supports the Weyl points and Fermi arcs. The topological features are further illustrated with the robust transport of edge states at an irregular boundary of the metamaterial.

## Introduction

Topological phases are states of matter that are characterized by integer quantities that remain constant under arbitrary continuous deformations of the system. A well understood form of the topological phase is the quantum Hall (QH) state^1^, which belongs to a topological class that breaks the time-reversal (TR) symmetry. A different topological class that preserves the TR symmetry is the quantum spin Hall (QSH) state^2,3^, in which the spin-orbit interaction is responsible for the topological character. The theoretical concepts developed in the QSH states were soon generalized to three-dimensional topological insulators^4^.

A remarkable feature of the topological phase is the emergence of gapless edge states that connect the valence band to the conduction band, which are protected by topology^5,6^. In the QH system, there exists a single edge mode that propagates unidirectionally at the boundary, which is called the *chiral* edge state^7^. The topological property of the QH system is characterized by the TKNN invariant or Chern number^8^. In the QSH system, there are a pair of edge modes that counterpropagate at a given edge, which are termed as the *helical* edge states^9^. Likewise, the topological property of the QSH system is determined by the Z_2_ invariant^10^ or spin Chern number^11^.

The novel concepts of topological phases have also been extended to photonic systems^12^, leading to the discovery of photonic QH states^13,14^ and photonic topological insulators^15–23^. Recently, topological semimetals that represent a new topological phase lacking a full band gap in the bulk states were proposed in photonic metamaterials^24–27^. They are three dimensional systems with multisheet Fermi (equifrequency) surfaces that embrace magnetic monopoles in the momentum (wave vector) space^24^. In parallel to the progress on photonic topological insulators, there has been a great deal of recent research into photonic Weyl semimetals^28–32^, where the Weyl points act as the source and sink of Berry curvature and are responsible for the nontrivial topological features^33^. The edge states in the photonic Weyl semimetal serve as the optical analogue of Fermi arcs that connect the Weyl points with opposite chiralities.

In the present study, we investigate the photonic topological phases in bianisotropic metamaterials characterized by a chirality tensor with zero trace. The electromagnetic duality allows for the hybrid modes to be decoupled into two subsystems when the duality condition^16,20,21^ is satisfied. By introducing the pseudospin states as the eigenfield basis, the hybrid modes are described by the spin-orbit Hamiltonians with spin 1^34–37^, which result in nonzero spin Chern numbers that characterize the topological properties. The QH states exist in the momentum gap when the longitudinal chirality component exceeds the permittivity, where the bulk modes are described by an ellipsoid and a two-sheeted hyperboloid. The QSH states are also present in the system when the chiral nihility occurs and the bulk modes are described by two degenerate hyperboloids. Considering the dispersion in the frequency domain, the underlying metamaterial is regarded as a photonic Weyl semimetal that supports the Weyl points and Fermi arcs.

The surface modes that connect two distinct topological phases are analytically formulated at the interface between vacuum and the metamaterial based on Maxwell’s boundary conditions. The surface modes are represented by dispersion surfaces in the frequency-wave vector space, which are reduced to surface-state arcs at a reference frequency. The topological features of the surface states are further demonstrated by the electromagnetic radiation excited by a point dipole placed at the interface. The surface waves propagate unidirectionally at an irregular boundary and are immune to backscattering.

## Results

### Bulk modes

Consider a bianisotropic metamaterial characterized by the constitutive relations:

1 $${\bf{D}}={\varepsilon }_{0}\underline{\varepsilon }{\bf{E}}+\sqrt{{\varepsilon }_{0}{\mu }_{0}}\underline{\xi }{\bf{H}},$$

2 $${\bf{B}}={\mu }_{0}\underline{\mu }{\bf{H}}+\sqrt{{\varepsilon }_{0}{\mu }_{0}}\underline{\zeta }{\bf{E}},$$

where $\underline{\varepsilon }$, $\underline{\mu }$, $\underline{\xi }$ and $\underline{\zeta }$ are the frequency-dependent permittivity, permeability, and magnetoelectric coupling tensors, respectively. Treating the combined electric field **E** = (*E*_*x*_, *E*_*y*_, *E*_*z*_) and magnetic field **H** = (*H*_*x*_, *H*_*y*_, *H*_*z*_) as a six-component vector, Maxwell’s equations for the time-harmonic fields (with the convention *e*^−*iωt*^) are written in matrix form as

3 $$(\begin{array}{cc}\omega \mathop{\varepsilon }\limits_{\_} & c{\bf{k}}\times \mathop{I}\limits_{\_}+\omega \mathop{\xi }\limits_{\_}\\ -c{\bf{k}}\times \mathop{I}\limits_{\_}+\omega \mathop{\zeta }\limits_{\_} & \omega \mathop{\mu }\limits_{\_}\end{array})(\begin{array}{c}{\bf{E}}\\ {{\bf{H}}}^{{\rm{^{\prime} }}}\end{array})=0,$$

where $\underline{I}$ is the 3 × 3 identity matrix, **H**′ =* η*_0_**H**, and ${\eta }_{0}=\sqrt{{\mu }_{0}/{\varepsilon }_{0}}$. Assume that the medium is reciprocal and the material tensors are uniaxial: $\underline{\varepsilon }={\rm{diag}}({\varepsilon }_{t},{\varepsilon }_{t},{\varepsilon }_{z})$, $\underline{\mu }={\rm{diag}}({\mu }_{t},{\mu }_{t},{\mu }_{z})$, and $\underline{\xi }=-\,\underline{\zeta }=i\underline{\gamma }={\rm{diag}}(i{\gamma }_{t},i{\gamma }_{t},i{\gamma }_{z})$, where *ε*_*n*_, *μ*_*n*_, and *γ*_*n*_ (*n* = *t*, *z*) are real quantities. The existence of a nontrivial solution of **E** and **H** requires that the determinant of the 6 × 6 matrix in Eq. (3) be zero, which gives the characteristic equation of the bulk modes as

4 $$\begin{array}{c}\frac{{k}_{t}^{4}}{{k}_{0}^{4}}({\varepsilon }_{t}^{2}-{\gamma }_{t}^{2})+({\varepsilon }_{z}{\mu }_{z}-{\gamma }_{z}^{2})[\frac{{k}_{z}^{4}}{{k}_{0}^{4}}-2\frac{{k}_{z}^{2}}{{k}_{0}^{2}}({\varepsilon }_{t}{\mu }_{t}+{\gamma }_{t}^{2})+{({\varepsilon }_{t}{\mu }_{t}-{\gamma }_{t}^{2})}^{2}]\\ \,+\,\frac{{k}_{t}^{2}}{{k}_{0}^{2}}[({\varepsilon }_{z}{\mu }_{t}+{\varepsilon }_{t}{\mu }_{z})(\frac{{k}_{z}^{2}}{{k}_{0}^{2}}-{\varepsilon }_{t}{\mu }_{t}+{\gamma }_{t}^{2})-2{\gamma }_{t}{\gamma }_{z}(\frac{{k}_{z}^{2}}{{k}_{0}^{2}}+{\varepsilon }_{t}{\mu }_{t}-{\gamma }_{t}^{2})]=0,\end{array}$$

where *k*_*t*_^2^ = *k*_*x*_^2^ + *k*_*y*_^2^ and *k*_0_ = *ω*/*c*. This is a bi-quadratic equation that incorporates the coupling between transverse electric and transverse magnetic modes.

We now assume that the metamaterial satisfies the duality condition: $\underline{\varepsilon }=\underline{\mu }$^16,20,21^. Equation (4) can then be factorized as

5 $$[\frac{{k}_{t}^{2}}{{\varepsilon }_{z}+{\gamma }_{z}}+\frac{{k}_{z}^{2}}{{\varepsilon }_{t}+{\gamma }_{t}}-({\varepsilon }_{t}+{\gamma }_{t}){k}_{0}^{2}][\frac{{k}_{t}^{2}}{{\varepsilon }_{z}-{\gamma }_{z}}+\frac{{k}_{z}^{2}}{{\varepsilon }_{t}-{\gamma }_{t}}-({\varepsilon }_{t}-{\gamma }_{t}){k}_{0}^{2}]=0,$$

which is a product of two quadratic equations. In this situation, the bulk modes are a combination of two decoupled modes with opposite signs of *γ*_*t*_ and *γ*_*z*_. In the neighborhood of a reference frequency *ω*_ref_, *ε*_*n*_ (*n* = *t*, *z*) can be approximated as ${\varepsilon }_{n}\approx {\varepsilon }_{n0}+{\frac{d{\varepsilon }_{n}}{d\omega }|}_{\omega ={\omega }_{{\rm{ref}}}}(\omega -{\omega }_{{\rm{ref}}})\equiv {\varepsilon }_{n0}+{\tilde{\varepsilon }}_{n}\delta \omega /{\omega }_{{\rm{ref}}}$, where ${\tilde{\varepsilon }}_{n}$ is positive definite^34^. We assume that the chirality parameters *γ*_*t*_ and *γ*_*z*_ vary smoothly around *ω*_ref_ and can be treated as constants as a first-order approximation. We further propose that the chirality tensor $\underline{\gamma }$ has a zero trace so that *γ*_*z*_ = −2*γ*_*t*_. In case *ε*_*t*_ = *ε*_*z*_, the underlying metamaterial is equivalent to the *pseudochiral* medium^38,39^ characterized by the magnetoelectric coupling tensors with zero diagonal and symmetric off-diagonal entries (see Methods A), which can be synthesized by the lattice of intersecting split-ring resonators^40^ or mutually perpendicular Ω-shaped metal wires^41^.

### Spin-orbit Hamiltonians

The electromagnetic duality of Maxwell’s equations dictates that the matrix in Eq. (3) holds a symmetric pattern when the duality condition $\underline{\varepsilon }=\underline{\mu }$ is satisfied. This enables us to rewrite the wave equations for the hybrid modes, defined by **F**^±^ = **E** ± *i***H**′, as

6 $$(\begin{array}{cc}{{\mathscr{H}}}_{0}({\bf{k}}{\boldsymbol{)}} & {\bf{0}}\\ {\bf{0}} & {{\mathscr{H}}}_{0}(\,-{\bf{k}}{\boldsymbol{)}}\end{array})(\begin{array}{c}{{\bf{F}}}^{{\boldsymbol{+}}}\\ {{\bf{F}}}^{{\boldsymbol{-}}}\end{array})=0,$$

where ${ {\mathcal H} }_{0}(\pm {\bf{k}})=\omega \underline{\varepsilon }\mp i(c{\bf{k}}\times \underline{I}+\omega \underline{\xi })$. Note that **F**^+^ and **F**^−^ are completely decoupled and determined by two subsystems (3 × 3 matrix) with a similar structure. In the isotropic case, where *ε*_*t*0_ = *ε*_*z*0_ ≡ *ε*, ${\tilde{\varepsilon }}_{t}={\tilde{\varepsilon }}_{z}\equiv \tilde{\varepsilon }$, and *γ*_*t*_ = *γ*_*z*_ ≡ *γ*, the wave equation can be rearranged as (see Methods B)

7 $${ {\mathcal H} }_{\pm }{\psi }_{\pm }-{d}_{\pm }{\psi }_{\pm }=\delta \omega {\psi }_{\pm }$$

by introducing the *pseudospin* state $${\psi }_{\pm }={U}^{-1}{\tilde{\psi }}_{\pm }$$, where $${\mathop{\psi }\limits^{ \sim }}_{\pm }={(\frac{\mp {F}_{x}^{\pm }+i{F}_{y}^{\pm }}{\sqrt{2}},{F}_{z},\frac{\pm {F}_{x}^{\pm }+i{F}_{y}^{\pm }}{\sqrt{2}})}^{T}$$ and $$U={\rm{diag}}$$$$(\sqrt{{\tilde{\varepsilon }}_{z}/{\tilde{\varepsilon }}_{t}},1,\sqrt{{\tilde{\varepsilon }}_{z}/{\tilde{\varepsilon }}_{t}})$$. In Eq. (7), $${d}_{\pm }={\omega }_{{\rm{ref}}}(\varepsilon /\tilde{\varepsilon }\pm \gamma )$$ and

8 $${ {\mathcal H} }_{+}=\alpha {\bf{k}}\cdot {\bf{S}},\,{ {\mathcal H} }_{-}=-\,\alpha {({\bf{k}}\cdot {\bf{S}})}^{\ast },$$

where $\alpha =c/\tilde{\varepsilon }$, ${\bf{k}}={k}_{x}\hat{x}+{k}_{y}\hat{y}+{k}_{z}\hat{z}$, ${\bf{S}}={S}_{x}\hat{x}+{S}_{y}\hat{y}+{S}_{z}\hat{z}$, and *S*_*n*_ (*n* = *x*, *y*, *z*) are the spin matrices for spin 1.

Note that Eq. (7) is formulated as an eigensystem with *δω* as the eigenvalue. The Hamiltonian ${ {\mathcal H} }_{\pm }$ in Eq. (8) represents the spin-orbit interaction **k** · **S** with spin 1, which is mathematically equivalent to the Hamiltonian of a magnetic dipole moment in the magnetic field^34,35^.

### Topological invariants

The topological properties of the spin-orbit Hamiltonians ${ {\mathcal H} }_{\pm }$ can be characterized by the topological invariants based on the eigenfields. For this purpose, we calculate the Berry flux over a closed surface *S*: ${k}_{x}^{2}+{k}_{y}^{2}+{k}_{z}^{2}={(\varepsilon \pm \gamma )}^{2}{k}_{0}^{2}$, corresponding to the bulk mode at the reference frequency *ω*_ref_ in the wave vector space. The eigensystem for the Hamiltonian ${ {\mathcal H} }_{\pm }$ in Eq. (8):

9 $${ {\mathcal H} }_{\pm }{\psi }_{\pm }^{\sigma }={\lambda }_{\pm }^{\sigma }{\psi }_{\pm }^{\sigma }$$

is solved to give the eigenvalues *λ*_±_^*σ*^ and eigenvectors *ψ*_±_^*σ*^ (*σ* = ±1, 0), based on which the Chern numbers are calculated to give (see Methods C)

10 $${C}_{\sigma }=2\sigma .$$

The nonzero *C*_*σ*_ (*σ* = ±1) characterize the topological properties of the system, where *σ* refers to the helicity (or handedness) of the pseudospin states. In particular, the helical edge states are topologically protected, which means that their existence is guaranteed by the difference in band topology on two sides of the interface. As long as the band gap is not closed, the topological invariants remain unchanged under arbitrary continuous deformations of the system. The topological properties of the isotropic medium will be retained when a certain anisotropy is included. For the anisotropic medium, the exact calculation of topological invariants can be obtained by the numerical integration of Berry curvatures^27^. In this system, the total Chern number $C=\sum _{\sigma }\,{C}_{\sigma }=0$ and the spin Chern number ${C}_{{\rm{spin}}}=\sum _{\sigma }\,\sigma {C}_{\sigma }=4$, which are consistent with the quantum spin Hall effect of light^42^.

If *γ* = 0, the Hamiltonian ${ {\mathcal H} }_{\pm }$ in Eq. (9) has degenerate eigenvalues: *λ*_+_^*σ*^ = *λ*_−_^*σ*^ ≡ *λ*_*σ*_. The eigensystem of the combined hybrid modes is written as

11 $$(\begin{array}{cc}{{\mathscr{H}}}_{+} & {\bf{0}}\\ {\bf{0}} & {{\mathscr{H}}}_{-}\end{array})(\begin{array}{c}{\psi }_{+}^{\sigma }\\ {\psi }_{-}^{\sigma }\end{array})={\lambda }_{\sigma }(\begin{array}{c}{\psi }_{+}^{\sigma }\\ {\psi }_{-}^{\sigma }\end{array}).$$

Another condition of degenerate eigenvalues occurs at *ε* = 0 (and *δω* = 0), which is known as the *chiral nihility*^43,44^. The eigenvalues of the Hamiltonian ${ {\mathcal H} }_{\pm }$ are given by *λ*_+_^*σ*^ = −*λ*_−_^*σ*^ ≡ *λ*_*σ*_ and the eigensystem is written as

12 $$(\begin{array}{cc}{{\mathscr{H}}}_{+} & {\bf{0}}\\ {\bf{0}} & {}_{-}{{\mathscr{H}}}_{-}\end{array})(\begin{array}{c}{\psi }_{+}^{\sigma }\\ {\psi }_{-}^{\sigma }\end{array})={\lambda }_{\sigma }(\begin{array}{c}{\psi }_{+}^{\sigma }\\ {\psi }_{-}^{\sigma }\end{array}).$$

In Eqs. (11) and (12), the combined Hamiltonian consists of two copies of the spin-orbit Hamiltonian with opposite helicity, which is characteristic of the Bernevig-Hughes-Zhang (BHZ) model for the QSH system^3^. In particular, the combined Hamiltonian is TR invariant under *T*_*p*_ (see Methods D):

13 $$({T}_{p}\otimes I){ {\mathcal H} }_{c}({\bf{k}}){({T}_{p}\otimes I)}^{-1}={ {\mathcal H} }_{c}(\,-\,{\bf{k}}),$$

where

14 $${{\mathscr{H}}}_{c}({\bf{k}})=(\begin{array}{cc}\alpha {\bf{k}}\cdot {\bf{S}} & {\bf{0}}\\ {\bf{0}} & \beta \alpha {({\bf{k}}\cdot {\bf{S}})}^{\ast }\end{array})$$

and *T*_*p*_ is the fermionic-like *pseudo* TR operator with *T*_*p*_^2^ = −1^20^. Here, *β* = −1 for *γ* = 0 (*ε* ≠ 0) and *β* = 1 for *ε* = 0 (*γ* ≠ 0) [cf. Equations. (8), (11), and (12)]. The pseudo TR symmetry of the combined Hamiltonian ${ {\mathcal H} }_{c}$ is crucial in determining the QSH phases in photonic systems of spin 1, which allows the coexistence of counterpropagating spin-polarized edge states as in electronic systems^12^. For a nonzero *γ* (and *ε* ≠ 0), ${ {\mathcal H} }_{+}$ and ${ {\mathcal H} }_{+}$ are no longer degenerate and the pseudo TR symmetry is not preserved.

### Surface modes

Let the *xz* plane be an interface between vacuum and the bianisotropic metamaterial characterized by *ε*_*t*_ = *ε*_*z*_ ≡ *ε*, *γ*_*t*_, and *γ*_*z*_. The characteristic equation of surface modes is formulated based on Maxwell’s boundary conditions: the continuity of tangential electric and magnetic field components at the interface, which is given by (see Methods E)

15 $$\begin{array}{c}{k}_{z}{k}_{0}^{2}\{{\alpha }_{+}{\alpha }_{-}({\alpha }_{-}{k}_{y}^{(3)}-{\alpha }_{+}{k}_{y}^{(4)})({k}_{y}^{(1)}{k}_{y}^{(2)}+{k}_{z}^{2})+{k}_{z}^{2}({\alpha }_{+}{k}_{y}^{(3)}-{\alpha }_{-}{k}_{y}^{(4)})\\ +\,{k}_{x}^{2}[{\alpha }_{-}({\alpha }_{+}^{2}-2){k}_{y}^{(4)}-{\alpha }_{+}({\alpha }_{-}^{2}-2){k}_{y}^{(3)}-4\varepsilon {\gamma }_{t}({k}_{y}^{(1)}+{k}_{y}^{(2)})]\}\\ -\,i{k}_{0}{k}_{x}{k}_{z}^{2}[2{\varepsilon }^{2}({k}_{y}^{(1)}{k}_{y}^{(2)}+{k}_{y}^{(3)}{k}_{y}^{(4)})+2({k}_{x}^{2}+{k}_{z}^{2})({\varepsilon }^{2}+{\gamma }_{t}^{2}-1)\\ -\,({k}_{y}^{(1)}+{k}_{y}^{(2)})({\alpha }_{+}{k}_{y}^{(3)}+{\alpha }_{-}{k}_{y}^{(4)})+2{\gamma }_{t}^{2}({k}_{y}^{(1)}{k}_{y}^{(2)}-{k}_{y}^{(3)}{k}_{y}^{(4)})]\\ +\,i{k}_{x}{k}_{0}^{3}\{{\alpha }_{+}{\alpha }_{-}^{2}[2{\alpha }_{+}({k}_{y}^{(1)}{k}_{y}^{(2)}+{k}_{z}^{2})-{k}_{y}^{(3)}({k}_{y}^{(1)}+{k}_{y}^{(2)})]\\ -\,2{k}_{z}^{2}({\varepsilon }^{2}+{\gamma }_{t}^{2})-{\alpha }_{+}{\alpha }_{-}{k}_{y}^{(4)}[{\alpha }_{+}({k}_{y}^{(1)}+{k}_{y}^{(2)})-2{k}_{y}^{(3)}]\}\\ +\,{\alpha }_{+}{\alpha }_{-}{k}_{z}{k}_{0}^{4}({\alpha }_{+}{k}_{y}^{(4)}-{\alpha }_{-}{k}_{y}^{(3)})+{k}_{z}^{3}{k}_{0}^{2}({\alpha }_{-}{k}_{y}^{(4)}-{\alpha }_{+}{k}_{y}^{(3)})=\mathrm{0,}\end{array}$$

where ${k}_{y}^{(1)}={k}_{y}^{(2)}=\sqrt{{k}_{0}^{2}-{k}_{x}^{2}-{k}_{z}^{2}}$ are the normal (to interface) wave vector components in vacuum, and ${k}_{y}^{(3)}=-\,\sqrt{{\beta }_{+}({\alpha }_{+}{k}_{0}^{2}-{k}_{z}^{2}/{\alpha }_{+})-{k}_{x}^{2}}$ and ${k}_{y}^{(4)}=-\,\sqrt{{\beta }_{-}({\alpha }_{-}{k}_{0}^{2}-{k}_{z}^{2}/{\alpha }_{-})-{k}_{x}^{2}}$ are the normal wave vector components in the bianisotropic medium, with *α*_±_ = *ε* ± *γ*_*t*_ and *β*_±_ = *ε* ± *γ*_*z*_. For the surface waves to exist on the vacuum side (*y* > 0), *k*_*y*_^(1,2)^ should be purely imaginary with a positive value, so that the waves decay exponentially away from the interface. On the bianisotropic medium side (*y* < 0), *k*_*y*_^(3)^ and *k*_*y*_^(4)^ should be purely imaginary with a negative value for a similar reason.

## Discussion

### Bulk modes

**Figure 1:**
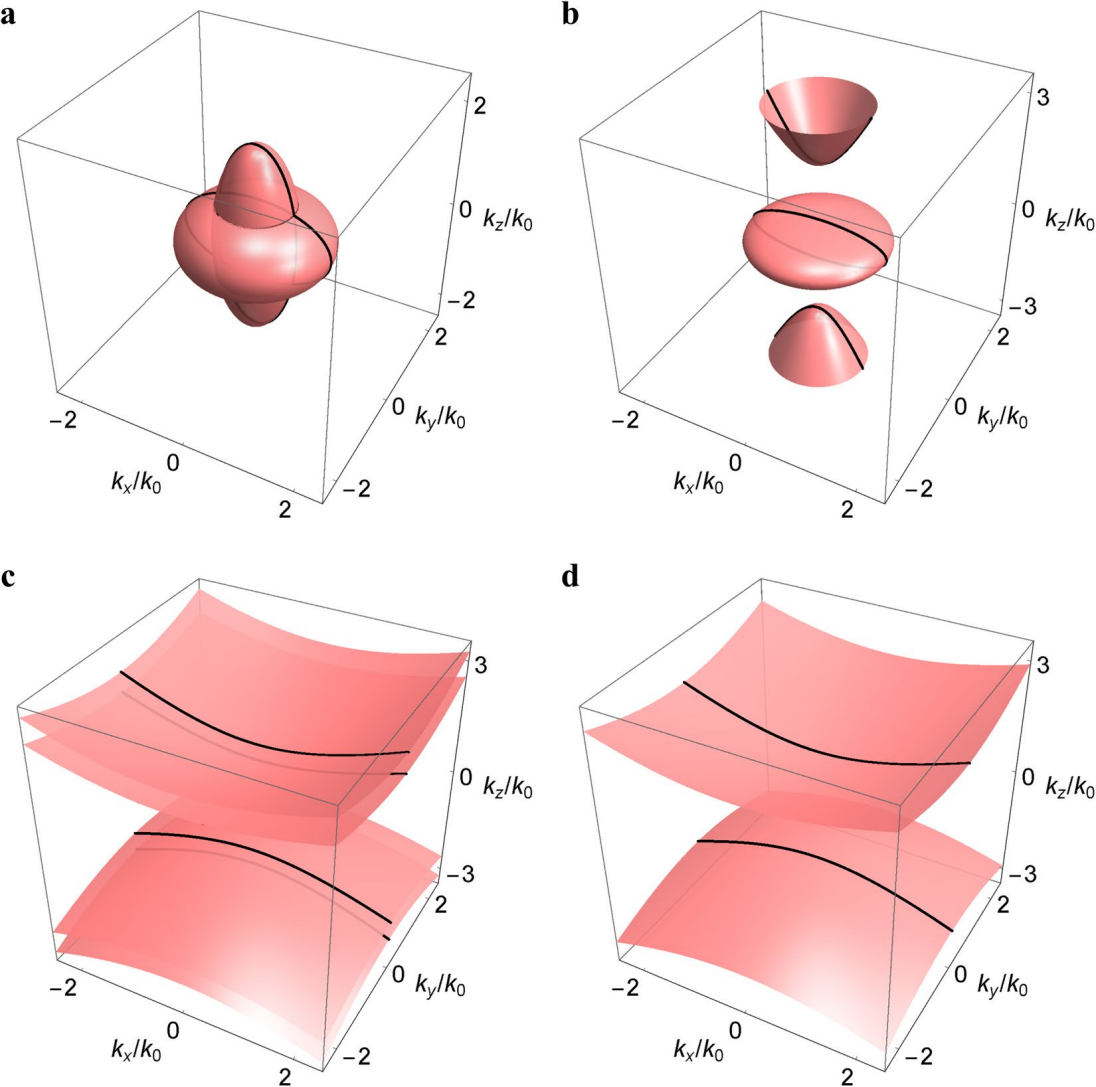
Equifrequency surfaces of the bulk modes in the wave vector space for the bianisotropic metamaterial based on Eq. (5) for (**a**) *ε* = 1.3 and *γ*_*z*_ = −2*γ*_*t*_ = −1 (**b**) *ε* = 1.3 and *γ*_*z*_ = −2*γ*_*t*_ = −1.5 (**c**) *ε* = 0.2 and *γ*_*z*_ = −2*γ*_*t*_ = −3 (**d**) *ε* = 0 and *γ*_*z*_ = −2*γ*_*t*_ = −3. Black lines are bulk modes at *k*_*y*_ = 0.
(II) If |*γ*_*z*_| < *ε* and *γ*_*t*_ < *ε*, the bulk modes are described by an ellipsoid and a two-sheeted hyperboloid [cf. Equation (5)]. Two band gaps (one for *k*_*z*_ > 0 and the other for *k*_*z*_ < 0) exist between three well separated bulk modes. The gap size Δ*k*_*z*_ = 2*γ*_*t*_*k*_0_ is determined by the band edges: *k*_*z*_/*k*_0_ = ±(*ε* ± *γ*_*t*_), between which the bulk modes do not exist. The equifrequency surfaces of the bulk modes are shown in Fig. 1(b) for *ε* = 1.3 and *γ*_*z*_ = −2*γ*_*t*_ = −1.5. In this phase, the present medium is equivalent to the chiral hyperbolic metamaterial^24^. The chirality components in the former (*γ*_*t*_*γ*_*z*_ < 0) play a similar role of the permittivity components in the latter (*ε*_*t*_*ε*_*z*_ < 0), giving rise to the same bulk dispersion.
(III) If |*γ*_*z*_| < *ε* and *γ*_*t*_ < *ε*, the bulk modes are described by two asymmetric two-sheeted hyperboloids [cf. Equation (5)] with a band gap Δ*k*_*z*_ = 2|*ε* − *γ*_*t*_|*k*_0_ in between. The equifrequency surfaces of the bulk modes are shown in Fig. 1(c) for *ε* = 0.2 and *γ*_*z*_ = −2*γ*_*t*_ = −3. In this phase, the present medium behaves like an anisotropic hyperbolic material.
(IV) If *ε* = 0 (and *μ* = 0), which is referred to as the chiral nihility^43,44^, the two hyperboloids are degenerate with an identical band gap Δ*k*_*z*_ = 2*γ*_*t*_*k*_0_. The equifrequency surfaces of the bulk modes are shown in Fig. 1(d) for *ε* = 0 and *γ*_*z*_ = −2*γ*_*t*_ = −3. In this phase, the present medium is equivalent to the double hyperbolic metamaterial^26^. The chirality components at nihility in the former (*ε* = 0 and *γ*_*t*_*γ*_*z*_ < 0) play a similar role of the permittivity and permeability components in the latter (*ε*_*t*_*ε*_*z*_ < 0, *μ*_*t*_*μ*_*z*_ < 0, and *γ*_*t*_ = *γ*_*z*_ = 0), giving rise to the same bulk dispersion.

Let the permittivity and transverse chirality component of the bianisotropic metamaterial be positive (*ε*_*t*_ = *ε*_*z*_ ≡ *ε* > 0, *γ*_*t*_ > 0, and *γ*_*z*_ < 0) at the reference frequency *ω*_ref_, without loss of generality. The bulk modes of the metamaterial can be classified into three phases:

(I) If |*γ*_*z*_| < *ε* and *γ*_*t*_ < *ε*, the bulk modes are described by two intersecting ellipsoids [cf. Equation (5)], with the major and minor axes given by $\sqrt{(\varepsilon \pm {\gamma }_{z})(\varepsilon \pm {\gamma }_{t})}{k}_{0}$ and (*ε* ± *γ*_*t*_)*k*_0_, respectively. The equifrequency surfaces of the bulk modes in the wave vector space are shown in Fig. 1(a) for *ε* = 1.3 and *γ*_*z*_ = −2*γ*_*t*_ = −1. In this phase, the present medium behaves like an ordinary anisotropic dielectric material.

### Edges modes

**Figure 2:**
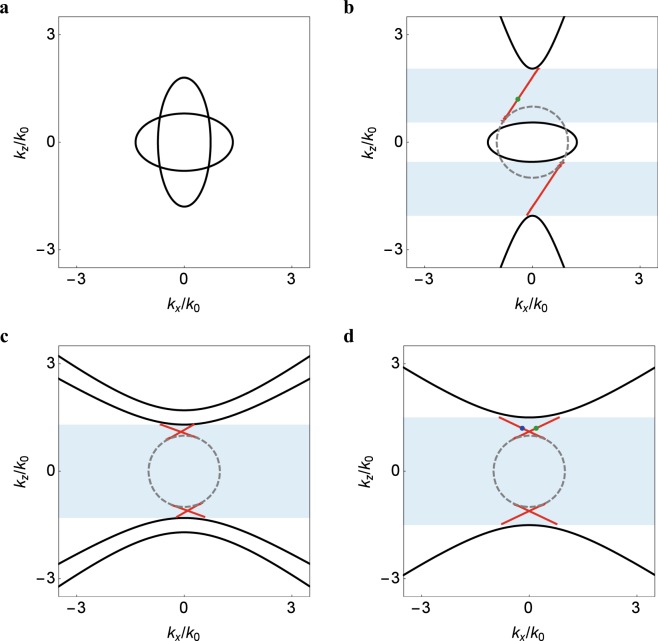
Surface modes at the interface between vacuum and the bianisotropic metamaterial based on Eq. (15) for (**a**) *ε* = 1.3 and *γ*_*z*_ = −2*γ*_*t*_ = −1 (**b**) *ε* = 1.3 and *γ*_*z*_ = −2*γ*_*t*_ = −1.5 (**c**) *ε* = 0.2 and *γ*_*z*_ = −2*γ*_*t*_ = −3 (**d**) *ε* = 0 and *γ*_*z*_ = −2*γ*_*t*_ = −3. Black curves are bulk modes of the metamaterial at *k*_*y*_ = 0. Gray dashed circle is dispersion surface of vacuum. Light blue regions correspond to band gaps.

Figure 2 shows the surface modes at the interface (*xz* plane) between vacuum and the bianisotropic metamaterial based on Eq. (15) for different phases stated in Sec. 3.1. For a small chirality parameter such that the system is in the phase (I), the bulk modes consist of two intersecting ellipsoids. As the common band gap between the bulk modes on both sides of the interface do not exist, there are no surface modes at the boundary of the metamaterial [cf. Fig. 2(a)]. For a larger chirality parameter such that the system is in the phase (II), one of the two ellipsoids is transformed into a two-sheeted hyperboloid. The change in topology of the bulk dispersion opens a nontrivial band gap, leading to the topological phase transition in the momentum space^45^. The topological invariants of the bulk modes are determined by integrating the Berry curvatures on the dispersion surface^27^, which give *C* = −2sgn(*γ*_*t*_) for the ellipsoid and *C* = sgn(*γ*_*t*_) for each sheet of the hyperboloid^40^.

There exists a single surface mode at the boundary of the metamaterial (adjacent to vacuum), which connects the hyperboloid dispersion of the metamaterial to the spherical dispersion (dashed circle) of vacuum [cf. Fig. 2(b)]. The surface mode lies inside the common band gap of the metamaterial and vacuum for either *k*_*z*_ > 0 or *k*_*z*_ < 0, which are antisymmetric with respect to the *k*_*x*_ axis. If the chirality parameter changes sign, the surface mode flips to the other side as a mirror reflection with respect to the *k*_*z*_ axis. In particular, the surface modes propagate unidirectionally at the boundary (either +*k*_*x*_ or −*k*_*x*_ axis), which is characteristic of the chiral edge states in the QH system, their existence being consistent with the bulk-edge correspondence^40^. In this situation, the bianisotropic medium can be regarded as a photonic analogue of the QH system. Note that the two subsystems in the combined Hamiltonian [cf. Equation (11)] are not degenerate in the presence of chirality, and the pseudo TR symmetry is broken in this phase. As there are no states available for backscattering, the chiral edge states are insensitive to disorder^5^.

For an even larger chirality parameter such that the system is in the phase (III), both ellipsoids are transformed into two-sheeted hyperboloids. There are a pair of surface modes at the boundary of the metamaterial (adjacent to vacuum) inside the band gap for either *k*_*z*_ > 0 or *k*_*z*_ < 0 [cf. Fig. 2(c)], which are in general asymmetric about the *k*_*z*_ axis. A particular situation occurs when the system is in the phase (IV), where the two hyperboloids are degenerate and the pair of surface modes is symmetric [cf. Fig. 2(d)]. The characteristic equation of surface modes [cf. Equation (15)] is simplified to

16 $${k}_{z}=\pm \frac{\sqrt{{\gamma }_{t}^{2}-1}}{\sqrt{1-{\gamma }_{t}{\gamma }_{z}}}{k}_{x}\pm \frac{\sqrt{{\gamma }_{t}^{2}-{\gamma }_{t}{\gamma }_{z}}}{\sqrt{1-{\gamma }_{t}{\gamma }_{z}}}{k}_{0},$$

which is a combination of four linear equations.

Note that the degenerate bulk dispersions correspond to two copies of the subsystems for the hybrid modes [cf. Equation (6)], which are represented by the spin-orbit Hamiltonians [cf. Equation (8)]. The topological invariants of the Hamiltonians are given by *C*_±_ = ±2 [cf. Equation (10)]. For a transition of the bulk dispersion from an ellipsoid to a two-sheeted hyperboloid, the closed surface in the wave vector space is cut open along the equator into two sheets, on which the Berry curvatures are flipped and the Chern numbers are equally split into half for each sheet: *C*_±_ = ±1^26^. In particular, the pair of surface modes with opposite spins counterpropagates at the same boundary, which is characteristic of the helical edge states in the QSH system, their existence being consistent with the bulk-edge correspondence. In this situation, the bianisotropic medium is regarded as a photonic analogue of the QSH system. The combined Hamiltonian respects the pseudo TR symmetry, leading to the topological protection of helical edge states in the photonic system.

### Weyl system

We now consider the explicit dispersion of the bianisotropic medium in the frequency domain. The Lorentz-type dispersive model, which is usually employed in the study of metamaterials, is adopted for the permittivity and permeability components: $\varepsilon ={\varepsilon }_{\infty }-{\omega }_{p}^{2}/({\omega }^{2}-{\omega }_{0}^{2})$ and *μ*_*n*_ = *μ*_*n*∞_ − Ω_*μn*_*ω*^2^/(*ω*^2^ − *ω*_0_^2^) (*n* = *t*, *z*)^26^, where *ω*_0_ is the resonance frequency of the resonators and *ω*_*p*_ is the effective plasma frequency of the medium. The chirality components are given by *γ*_*n*_ = Ω_*γn*_*ωω*_*p*_/(*ω*^2^ − *ω*_0_^2^), where Ω_*γn*_^2^ = Ω_*μn*_^46,47^. Such a dispersion guarantees that the energy density of the present medium is positive definite (see Methods F). In the present study, the bianisotropic response can be modelled by metallic helices oriented along three perpendicular directions^39^. In particular, the handedness of the helix in *z* direction is flipped so that the corresponding chirality component changes sign. The size of *z*-directed helix is adjusted in order to satisfy the traceless condition of the chirality tensor (2*γ*_*t*_ + *γ*_*z*_ = 0).

**Figure 3:**
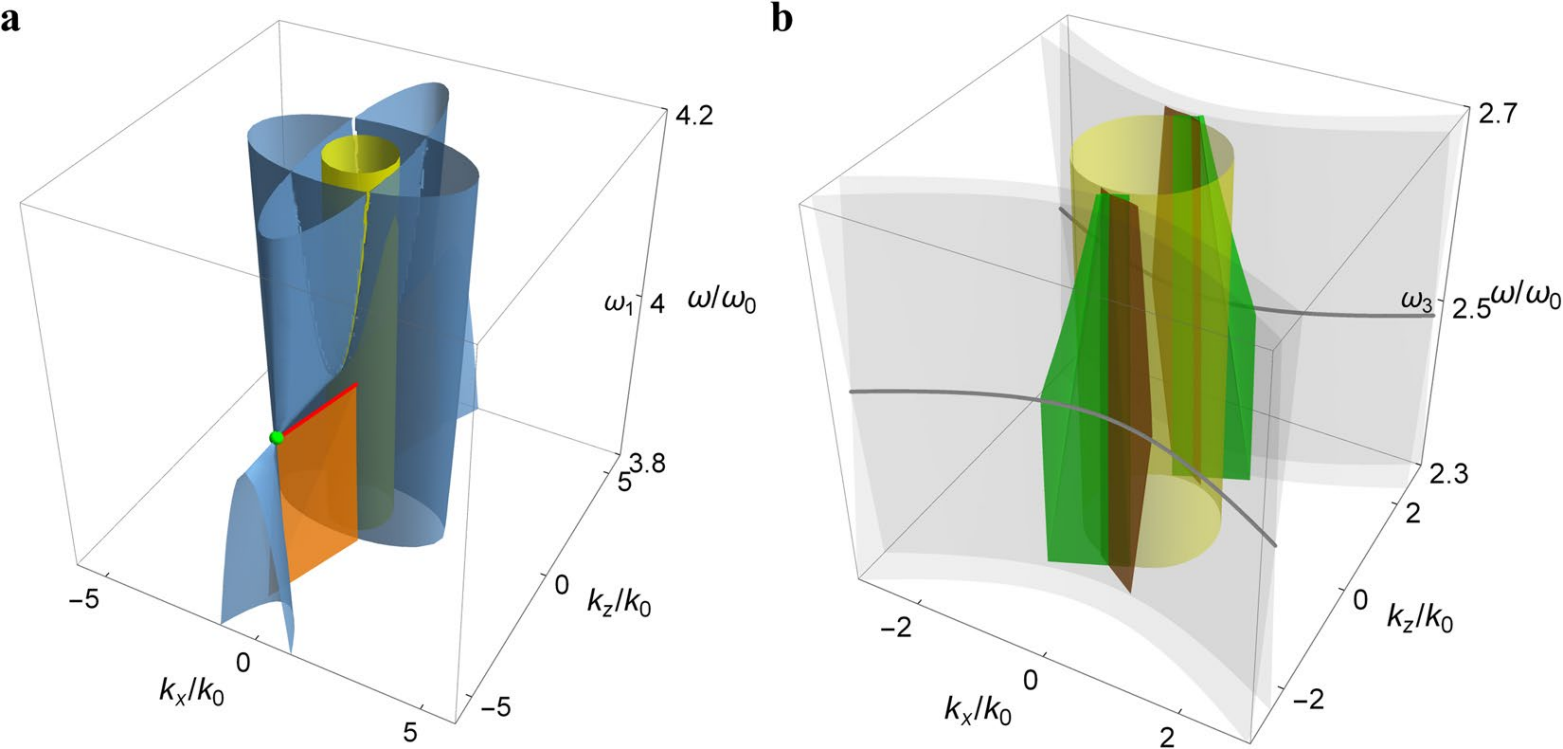
Bulk and surface modes in the frequency-wave vector space for the bianisotropic metamaterial with (**a**) *ω*_*p*_/*ω*_0_ = 6, *ε*_∞_ = 5.4, *μ*_*t*∞_ = 3.938, *μ*_*z*∞_ = 6.75, Ω_*μt*_ = 0.879, Ω_*μz*_ = 3.516, Ω_*γt*_ = 0.938, and Ω_*γz*_ = −1.875 (**b**) *ω*_*p*_/*ω*_0_ = 3.75, *ε*_∞_ = 2.679, *μ*_*t*∞_ = 0.84, *μ*_*z*∞_ = 3.36, Ω_*μt*_ = 0.706, Ω_*μz*_ = 2.822, Ω_*γt*_ = 0.84, and Ω_*γz*_ = −1.68. Blue surfaces are bulk modes. Orange and green surfaces are surface modes. Yellow cylinder is dispersion surface of vacuum. Green dot is the Weyl point. Red line is the Fermi arc. Gray lines are bulk modes for *ε* = 0. In (**b**), bulk modes are made transparent for a clear view of surface modes.

Figure 3(a) shows the bulk and surface modes of the bianisotropic metamaterial in the frequency-wave vector space. In the frequency range: *ω* > *ω*_1_, the bianisotropic medium is in the phase (I) as a dielectric system, where *ω*_1_ is the frequency across which the transition between the phase (I) (two ellipsoids) and the phase (II) (an ellipsoid and a two-sheeted hyperboloid) occurs, that is, *ε*(*ω*_1_) = *μ*_*n*_(*ω*_1_) = |*γ*_*z*_(*ω*_1_)|. It is noted that one ellipsoid in (I) and the hyperboloid in (II) touch at a pair of *Weyl points* symmetrically displaced on the *k*_*z*_ axis: (*k*_*t*_, *k*_*z*_) = (0, ±(*γ*_*t*_ − *γ*_*z*_)*k*_0_) [cf. green dot in Fig. 3(a)], which resembles the crossing of valence and conduction bands in the Weyl semimetal^33^. The frequency dispersion represents a tilted Weyl cone^29^, corresponding to the transition between type I Weyl points with spherical or ellipsoid dispersion surfaces and type II Weyl points with hyperbolic dispersion surfaces^48^. The surface modes (between the bianisotropic medium and vacuum) at the Weyl point frequency form the Fermi-arc-like edge states that connect the Weyl points [cf. red line in Fig. 3(a)]. The characteristic equation of surface modes [cf. Equation(15)] at this frequency is simplified to

17 $${k}_{z}={\rm{sgn}}({\gamma }_{t})\sqrt{{({\gamma }_{t}-{\gamma }_{z})}^{2}-1}{k}_{x}\pm ({\gamma }_{t}-{\gamma }_{z}){k}_{0},$$

which is a combination of two linear equations.

In the frequency range: *ω*_2_ < *ω* < *ω*_1_, the bianisotropic medium is in the phase (II) as a QH system, where *ω*_2_ is the frequency across which the transition between the phase (II) (an ellipsoid and a two-sheeted hyperboloid) and the phase (III) (two asymmetric hyperboloids) occurs, that is, *ε*(*ω*_2_) = *μ*_*n*_(*ω*_2_) = *γ*_*t*_(*ω*_2_). In this range, the surface modes are approximate flat surfaces that slightly change their orientations with the frequency [cf. orange surface in Fig. 3(a)]. In the frequency range: *ω* < *ω*_2_, the bianisotropic medium is in the phase (III) as a bi-hyperbolic system. The surface modes are a pair of approximate flat surfaces in a somewhat smaller frequency range, where a common band gap of the metamaterial and vacuum exists. In this range, the surface modes are intersecting approximate flat surfaces [orange and green surfaces in Fig. 3(b)]. At a particular frequency *ω* = *ω*_3_, the bianisotropic medium is in the phase (IV) as a QSH system, where *ω*_3_ is the frequency at which the chiral nihility occurs, that is, *ε*(*ω*_3_) = *μ*_*n*_(*ω*_3_) = 0. The two bulk modes are degenerate [cf. thick gray curves in Fig. 3(b)], which form the *nodal lines*^49^ at the chiral nihility frequency.

**Figure 4:**
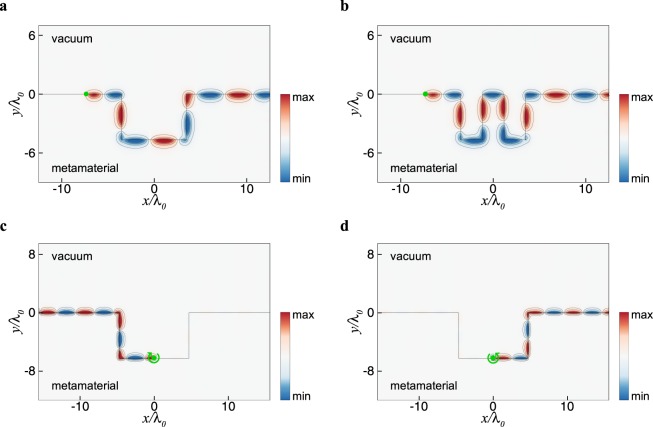
Surface wave propagation at the interface between vacuum and the bianisotropic metamaterial excited by a dipole source with *k*_*z*_/*k*_0_ = 1.2 for (**a**,**b**) *ε* = 1.3 and *γ*_*z*_ = −2*γ*_*t*_ = −1.5 (**c**,**d**) *ε* = 0 and *γ*_*z*_ = −2*γ*_*t*_ = −3. In (**c**,**d**), green curved arrows indicate the handedness of the dipole.

Finally, the topological features of edge states are illustrated with the surface wave propagation at the interface between vacuum and the metamaterial^24,27^, as shown in Fig. 4. A dipole source is placed at the interface (marked by dot symbol) to excite the surface mode in the band gap of the metamaterial but outside the light cone of vacuum, so that the fields are evanescent on both sides (see Methods G). In Fig. 4(a,b), the surface modes are excited at *k*_*z*_/*k*_0_ = 1.2 [cf. green dot in Fig. 2(b)], which propagate unidirectionally along an irregular boundary and are able to bend around sharp corners without backscattering. The propagation direction is reversed when the sign of chirality parameter is changed, showing the chiral nature of edge states in the phase (II), where the metamaterial behaves like a QH system. In Fig. 4(c,d), the surface modes are excited at the same *k*_*z*_, which correspond to a pair of *k*_*x*_’s [cf. green and blue dots in Fig. 2(d)]. In particular, the surface waves counterpropagate at the boundary for different handednesses of circular or elliptical polarization, which are immune to backscattering from disorder. The robust transport of the surface modes exhibits the helical nature of spin-polarized edge states in the phase (IV), where the metamaterial is regarded a QSH system.

In conclusion, we have investigated the photonic topological phases in bianisotropic metamaterials characterized by a chirality tensor with zero trace. The underlying medium is regarded as a photonic Weyl semimetal that supports the Weyl points and Fermi arcs. In particular, the photonic QH and QSH states exist in the momentum gap of the bulk modes, which are analytically formulated based on Maxwell’s equations. The topological properties are described by the spin-orbit Hamiltonians and characterized by the nozero topological invariants. The topological features are further illustrated with the robust transport of edge states at an irregular boundary of the metamaterial.

## Methods

### Bianisotropic and pseudochiral media

Consider a pseudochiral medium characterized by the magnetoelectric coupling tensor $\underline{\xi }^{\prime} =-\,\underline{\zeta }^{\prime}$ with zero diagonal and symmetric off-diagonal entries as

18 $${\mathop{\xi }\limits_{\_}}^{{\rm{^{\prime} }}}=(\begin{array}{ccc}0 & -i\gamma  & -i\gamma \\ -i\gamma  & 0 & -i\gamma \\ -i\gamma  & -i\gamma  & 0\end{array}).$$

The above matrices can be diagonalized through a similarity transformation:

19 $$\mathop{\xi }\limits_{\_}={P}^{-1}{\mathop{\xi }\limits_{\_}}^{{\rm{^{\prime} }}}P=(\begin{array}{ccc}i\gamma  & 0 & 0\\ 0 & i\gamma  & 0\\ 0 & 0 & -2i\gamma \end{array}),$$

where

20 $$P=(\begin{array}{ccc}-1 & -1 & 1\\ 0 & 1 & 1\\ 1 & 0 & 1\end{array})$$

is the transformation matrix formed by the column eigenvectors of $\underline{\xi }^{\prime}$. Note that $\underline{\varepsilon }^{\prime} ={\rm{diag}}(\varepsilon ,\varepsilon ,\varepsilon )$ and $\underline{\mu }^{\prime} ={\rm{diag}}(\mu ,\mu ,\mu )$ remain unchanged under the transformation: $\underline{\varepsilon }={P}^{-1}\underline{\varepsilon }^{\prime} P={\rm{diag}}(\varepsilon ,\varepsilon ,\varepsilon )$ and $\underline{\mu }={P}^{-1}\underline{\mu }^{\prime} P={\rm{diag}}(\mu ,\mu ,\mu )$. The above transformation shows that the pseudochiral medium characterized by the zero-diagonal $\underline{\xi }^{\prime}$ (${\xi ^{\prime} }_{ii}=0$) in the wave vector space ${{\bf{k}}}^{{\rm{^{\prime} }}}=({k}_{x}^{{\rm{^{\prime} }}},{k}_{y}^{{\rm{^{\prime} }}},{k}_{z}^{{\rm{^{\prime} }}})$ is equivalent to the bianisotropic medium characterized by the traceless $\underline{\xi }$ (${\sum }_{i}\,{\xi }_{ii}=0$) in the space ***k*** = (*k*_*x*_, *k*_*y*_, *k*_*z*_), where **k**′ = *P***k**. In the present study, the magnetoelectric coupling matrix contains nonzero diagonal elements with opposite signs [cf. Equation (19)], indicating that the waves propagating along the optical axis (*z*) and its transverse directions (*x* and *y*) exhibit opposite optical activities in the underlying medium^40^.

### Spin-orbit Hamiltonians

The wave equation for the hybrid modes **F**^±^ = **E** ± *i***H**′ in Eq. (6) can be rewritten as

21 $${\tilde{ {\mathcal H} }}_{\pm }{\tilde{\psi }}_{\pm }={\tilde{D}}_{\pm }{\tilde{\psi }}_{\pm },$$

where

22 $${\mathop{{\mathscr{H}}}\limits^{ \sim }}_{\pm }=c(\begin{array}{ccc}\pm {k}_{z} & \frac{\pm {k}_{x}-i{k}_{y}}{\sqrt{2}} & 0\\ \frac{\pm {k}_{x}+i{k}_{y}}{\sqrt{2}} & 0 & \frac{\pm {k}_{x}-i{k}_{y}}{\sqrt{2}}\\ 0 & \frac{\pm {k}_{x}+i{k}_{y}}{\sqrt{2}} & \mp {k}_{z}\end{array}),$$

23 $${\mathop{D}\limits^{ \sim }}_{\pm }=\omega (\begin{array}{ccc}{\varepsilon }_{t}\pm {\gamma }_{t} & 0 & 0\\ 0 & {\varepsilon }_{z}\pm {\gamma }_{z} & 0\\ 0 & 0 & {\varepsilon }_{t}\pm {\gamma }_{t}\end{array}),$$

and ${\mathop{\psi }\limits^{ \sim }}_{\pm }={(\frac{\mp {F}_{x}+i{F}_{y}}{\sqrt{2}},{F}_{z},\frac{\pm {F}_{x}+i{F}_{y}}{\sqrt{2}})}^{T}$ is the basis of the *pseudospin* states that include a *π*/2 phase difference between the transverse field components (with respect to the optical axis of the medium)^34^. In the neighborhood of a reference frequency *ω*_ref_, *ε*_*n*_ (*n* = *t*, *z*) can be approximated as ${\varepsilon }_{n}\approx {\varepsilon }_{n0}+{\frac{d{\varepsilon }_{n}}{d\omega }|}_{\omega ={\omega }_{{\rm{ref}}}}(\omega -{\omega }_{{\rm{ref}}})\equiv {\varepsilon }_{n0}+{\tilde{\varepsilon }}_{n}\delta \omega /{\omega }_{{\rm{ref}}}$, where ${\tilde{\varepsilon }}_{n}$ is positive definite^34^. Equation (21) is rearranged as

24 $${ {\mathcal H} }_{\pm }{\psi }_{\pm }-{D}_{\pm }{\psi }_{\pm }=\delta \omega {\psi }_{\pm },$$

where

25 $${{\mathscr{H}}}_{\pm }=\frac{c}{\sqrt{{\mathop{\varepsilon }\limits^{ \sim }}_{t}{\mathop{\varepsilon }\limits^{ \sim }}_{z}}}(\begin{array}{ccc}\pm \sqrt{\frac{{\mathop{\varepsilon }\limits^{ \sim }}_{z}}{{\mathop{\varepsilon }\limits^{ \sim }}_{t}}}{k}_{z} & \frac{\pm {k}_{x}-i{k}_{y}}{\sqrt{2}} & 0\\ \frac{\pm {k}_{x}+i{k}_{y}}{\sqrt{2}} & 0 & \frac{\pm {k}_{x}-i{k}_{y}}{\sqrt{2}}\\ 0 & \frac{\pm {k}_{x}+i{k}_{y}}{\sqrt{2}} & \mp \sqrt{\frac{{\mathop{\varepsilon }\limits^{ \sim }}_{z}}{{\mathop{\varepsilon }\limits^{ \sim }}_{t}}}{k}_{z}\end{array}),$$

26 $${D}_{\pm }={\omega }_{{\rm{r}}{\rm{e}}{\rm{f}}}(\begin{array}{ccc}\frac{{\varepsilon }_{t0}}{{\mathop{\varepsilon }\limits^{ \sim }}_{t}}\pm {\gamma }_{t} & 0 & 0\\ 0 & \frac{{\varepsilon }_{t0}}{{\mathop{\varepsilon }\limits^{ \sim }}_{z}}\pm {\gamma }_{z} & 0\\ 0 & 0 & \frac{{\varepsilon }_{t0}}{{\mathop{\varepsilon }\limits^{ \sim }}_{t}}\pm {\gamma }_{t}\end{array}),$$

and ${\psi }_{\pm }={U}^{-1}{\tilde{\psi }}_{\pm }$ with $U={\rm{diag}}(\sqrt{{\tilde{\varepsilon }}_{z}/{\tilde{\varepsilon }}_{t}},1,\sqrt{{\tilde{\varepsilon }}_{z}/{\tilde{\varepsilon }}_{t}})$. In the isotropic case, where *ε*_*t*0_ = *ε*_*z*0_ ≡ *ε*, ${\tilde{\varepsilon }}_{t}={\tilde{\varepsilon }}_{z}\equiv \tilde{\varepsilon }$, and *γ*_*t*_ = *γ*_*z*_ ≡ *γ*, Eq. (24) is simplified to

27 $${ {\mathcal H} }_{\pm }{\psi }_{\pm }-{d}_{\pm }{\psi }_{\pm }=\delta \omega {\psi }_{\pm },$$

where $${d}_{\pm }={\omega }_{{\rm{ref}}}(\varepsilon /\tilde{\varepsilon }\pm \gamma )$$ and

28 $${ {\mathcal H} }_{+}=\alpha {\bf{k}}\cdot {\bf{S}},\,{ {\mathcal H} }_{-}=-\,\alpha {({\bf{k}}\cdot {\bf{S}})}^{\ast }$$

with $$\alpha =c/\tilde{\varepsilon }$$, $${\bf{k}}={k}_{x}\hat{x}+{k}_{y}\hat{y}+{k}_{z}\hat{z}$$, $${\bf{S}}={S}_{x}\hat{x}+{S}_{y}\hat{y}+{S}_{z}\hat{z}$$, and

29 $${S}_{x}=\frac{1}{\sqrt{2}}(\begin{array}{ccc}0 & 1 & 0\\ 1 & 0 & 1\\ 0 & 1 & 0\end{array}),\,{S}_{y}=\frac{1}{\sqrt{2}}(\begin{array}{ccc}0 & -i & 0\\ i & 0 & -i\\ 0 & i & 0\end{array}),\,{S}_{z}=(\begin{array}{ccc}1 & 0 & 0\\ 0 & 0 & 0\\ 0 & 0 & -1\end{array})$$

being the spin matrices for spin 1.

### Topological invariants

In terms of the spherical coordinates, the Hamiltonian ${ {\mathcal H} }_{\pm }$ [cf. Equation (28)] is rewritten as

30 $${{\mathscr{H}}}_{\pm }=\pm \,\frac{|{d}_{\pm }|}{\sqrt{2}}(\begin{array}{ccc}\sqrt{2}\,\cos \,\theta  & \sin \,\theta {e}^{\mp i\phi } & 0\\ \sin \,\theta {e}^{\pm i\phi } & 0 & \sin \,\theta {e}^{-i\phi }\\ 0 & \sin \,\theta {e}^{\pm i\phi } & \mp \sqrt{2}\,\cos \,\theta \end{array}),$$

where *k*_*x*_ = *a*sin*θ*cos*ϕ*, *k*_*y*_ = *a*sinθsin*ϕ*, and *k*_*z*_ = *a*cos*θ* with *a* = |*ε* ± *γ*|*k*_0_. Here, *θ* and *ϕ* are the polar and azimuthal angles, respectively, on the closed surface *S*: ${k}_{x}^{2}+{k}_{y}^{2}+{k}_{z}^{2}={(\varepsilon \pm \gamma )}^{2}{k}_{0}^{2}$, corresponding to the bulk mode at the reference frequency *ω*_ref_ in the wave vector space. The eigensystem for the Hamiltonian ${ {\mathcal H} }_{\pm }$:

31 $${ {\mathcal H} }_{\pm }{\psi }_{\pm }^{\sigma }={\lambda }_{\pm }^{\sigma }{\psi }_{\pm }^{\sigma }$$

is solved to give the eigenvalues *λ*_±_^*σ*^ = |*d*_±_|*σ* (*σ* = ±1, 0) and the normalized eigenvectors as

32 $${\psi }_{\pm }^{\sigma }=\frac{1}{2}(\begin{array}{c}\sigma {e}^{\mp 2i\phi }(\sigma +\,\cos \,\theta )\\ \sigma \sqrt{2}{e}^{\mp i\phi }\,\sin \,\theta \\ 1-\sigma \,\cos \,\theta \end{array})\,(\sigma =\pm \,1),$$

33 $${\psi }_{\pm }^{\sigma }=\frac{1}{\sqrt{2}}(\begin{array}{c}-{e}^{\mp 2i\phi }\,\sin \,\theta \\ \sqrt{2}{e}^{\mp i\phi }\,\cos \,\theta \\ \sin \,\theta \end{array})\,(\sigma =0).$$

Note here that the eigenvalue *λ*_±_^*σ*^ is related to *δω* in Eq. (7) as *λ*_±_^*σ*^ = *d*_±_ + *δω*. Based on Eqs. (32) and (33), the Berry connections ${{\bf{A}}}_{\sigma }=-\,i\langle {\psi }_{\pm }^{\sigma }|\nabla {\psi }_{\pm }^{\sigma }\rangle$ are obtained as

34 $${{\bf{A}}}_{\sigma }=-\,\frac{1}{r}\,\cot \,\frac{\theta }{2}\hat{\phi }\,(\sigma =1),$$

35 $${{\bf{A}}}_{\sigma }=-\,\frac{1}{r}\,\tan \,\frac{\theta }{2}\hat{\phi }\,(\sigma =-\,1),$$

36 $${{\bf{A}}}_{\sigma }=-\,\frac{1}{r}\csc \theta \hat{\phi }\,(\sigma =0).$$

The Berry curvatures **F**_*σ*_ = ∇ × **A**_*σ*_ are then given by

37 $${{\bf{F}}}_{\sigma }=\sigma \frac{\hat{r}}{{r}^{2}}\,(\sigma =\pm \,1,0).$$

Integrating over the closed sphere *S*, the Chern numbers ${C}_{\sigma }=\frac{1}{2\pi }{\int }_{S}\,{{\bf{F}}}_{\sigma }\cdot ds$ are calculated to give

38 $${C}_{\sigma }=2\sigma \,(\sigma =\pm 1,0).$$

### Pseudo time-reversal symmetry

The Hamiltonian for Maxwell’s equations [cf. Equation (3)] in a lossless and reciprocal medium is time-reversal (TR) invariant under *T*_*b*_, that is,

39 $$({T}_{b}\otimes I){ {\mathcal H} }_{m}({\bf{k}}){({T}_{b}\otimes I)}^{-1}={ {\mathcal H} }_{m}(-\,{\bf{k}}),$$

where

40 $${{\mathscr{H}}}_{m}({\bf{k}})=(\begin{array}{cc}\omega \mathop{\varepsilon }\limits_{\_} & c{\bf{k}}\times \mathop{I}\limits_{\_}+\omega \mathop{\xi }\limits_{\_}\\ -c{\bf{k}}\times \mathop{I}\limits_{\_}+\omega \mathop{\zeta }\limits_{\_} & \omega \mathop{\mu }\limits_{\_}\end{array}),$$

*T*_*b*_ = *σ*_*z*_*K* (with *T*_*b*_^2^ = 1) is the bosonic TR operator for photons, and *K* is the complex conjugation^12^. The Hamiltonian ${ {\mathcal H} }_{m}$, however, is not TR invariant under *T*_*f*_, that is, (*T*_*f*_ ⊗ *I*)${ {\mathcal H} }_{m}$(**k**)(*T*_*f*_ ⊗ *I*)^−1^ ≠ ${ {\mathcal H} }_{m}$(−**k**), where *T*_*f*_ = *iσ*_*y*_*K* (with *T*_*f*_^2^ = −1) is the fermionic TR operator for electrons^12^. The combined Hamiltonian for the hybrid modes with the duality condition: $\underline{\varepsilon }=\underline{\mu }$, nevertheless, is TR invariant under *T*_*p*_, that is,

41 $$({T}_{p}\otimes I){ {\mathcal H} }_{c}({\bf{k}}){({T}_{p}\otimes I)}^{-1}={ {\mathcal H} }_{c}(\,-\,{\bf{k}}),$$

where

42 $${{\mathscr{H}}}_{c}({\bf{k}})=(\begin{array}{cc}\alpha {\bf{k}}\cdot {\bf{S}} & {\bf{0}}\\ {\bf{0}} & \beta \alpha {({\bf{k}}\cdot {\bf{S}})}^{\ast }\end{array})$$

and *T*_*p*_ is the fermionic-like *pseudo* TR operator having the same form of *T*_*f*_. Here, *β* = −1 for *γ* = 0 (*ε* ≠ 0) and *β* = 1 for *ε* = 0 (*γ* ≠ 0) [cf. Equations (8), (11), and (12)]. The pseudo TR operator *T*_*p*_ is inspired by noticing that **E** + *i***H**′ ↔ **E** − *i***H**′ during the TR operation. The pseudo TR operator is thus defined as *T*_*p*_ = *T*_*b*_*σ*_*x*_ = *σ*_*z*_*Kσ*_*x*_ = *iσ*_*y*_*K* with *T*_*p*_^2^ = −1^20^. Here, *σ*_*x*_ = (0, 1; 1, 0), *σ*_*y*_ = (0, −*i*; *i*, 0), and *σ*_*z*_ = diag(1, −1) are the Pauli matrices.

### Surface wave equation

According to Maxwell’s equations, the eigenfields on either side of the interface (*y* = 0) are given by the nontrivial solutions of **E** and **H** [cf. Equation (3)] or the *null space* of ${ {\mathcal H} }_{m}$ [cf. Equation (40)]. On the vacuum, side (*y* > 0), we have

43 $${{\bf{H}}}^{\mathrm{(1)}}=\frac{1}{{\eta }_{0}{k}_{0}}({k}_{z},\,\mathrm{0,}-\,{k}_{x}),\,{{\bf{E}}}^{\mathrm{(1)}}=\frac{1}{{k}_{0}^{2}}({k}_{x}{k}_{y}^{\mathrm{(1)}},-\,{k}_{x}^{2}-{k}_{z}^{2},{k}_{y}^{\mathrm{(1)}}{k}_{z}),$$

44 $${{\bf{H}}}^{\mathrm{(2)}}=\frac{1}{{\eta }_{0}{k}_{0}}({k}_{y}^{\mathrm{(2)}},-\,{k}_{x}\mathrm{,0}),\,{{\bf{E}}}^{\mathrm{(2)}}=-\,\frac{1}{{k}_{0}^{2}}({k}_{x}{k}_{z},{k}_{y}^{\mathrm{(2)}}{k}_{z},{k}_{z}^{2}-{k}_{0}^{2}),$$

where ${k}_{y}^{(1)}={k}_{y}^{(2)}=\sqrt{{k}_{0}^{2}-{k}_{x}^{2}-{k}_{z}^{2}}$ are the normal (to interface) wave vector components in vacuum, and the superscripts (1) and (2) refer to two independent polarization. On the bianisotropic medium side (*y* < 0), the eigenfields are given by

45 $${{\bf{H}}}^{(3)}=\frac{1}{{\eta }_{0}{k}_{0}^{2}}({k}_{x}{k}_{z}-i{\alpha }_{+}{k}_{y}^{(3)}{k}_{0},i{\alpha }_{+}{k}_{x}{k}_{0}+{k}_{y}^{(3)}{k}_{z},{k}_{z}^{2}-{\alpha }_{+}^{2}{k}_{0}^{2}),$$

46 $${{\bf{E}}}^{(3)}=\frac{i}{{k}_{0}^{2}}({k}_{x}{k}_{z}-i{\alpha }_{+}{k}_{y}^{(3)}{k}_{0},i{\alpha }_{+}{k}_{x}{k}_{0}+{k}_{z}{k}_{y}^{(3)},{k}_{z}^{2}-{\alpha }_{+}^{2}{k}_{0}^{2}),$$

47 $${{\bf{H}}}^{(4)}=\frac{1}{{\eta }_{0}{k}_{0}^{2}}({k}_{x}{k}_{z}+i{\alpha }_{-}{k}_{y}^{(4)}{k}_{0},{k}_{y}^{(4)}{k}_{z}-i{\alpha }_{-}{k}_{x}{k}_{0},{k}_{z}^{2}-{\alpha }_{-}^{2}{k}_{0}^{2}),$$

48 $${{\bf{E}}}^{(4)}=-\,\frac{i}{{k}_{0}^{2}}({k}_{x}{k}_{z}+i{\alpha }_{-}{k}_{y}^{(4)}{k}_{0},{k}_{z}{k}_{y}^{(4)}-i{\alpha }_{-}{k}_{x}{k}_{0},{k}_{z}^{2}-{\alpha }_{-}^{2}{k}_{0}^{2}),$$

where ${k}_{y}^{(3)}=-\,\sqrt{{\beta }_{+}({\alpha }_{+}{k}_{0}^{2}-{k}_{z}^{2}/{\alpha }_{+})-{k}_{x}^{2}}$ and ${k}_{y}^{(4)}=-\,\sqrt{{\beta }_{-}({\alpha }_{-}{k}_{0}^{2}-{k}_{z}^{2}/{\alpha }_{-})-{k}_{x}^{2}}$ are the normal wave vector components in the bianisotropic medium, with *α*_±_ = *ε* ± *γ*_*t*_ and *β*_±_ = *ε* ± *γ*_*z*_, and the superscripts (3) and (4) refer to two independent polarizations. Note that the eigenfields in Eqs. (43)–(48) share the common tangential wave vector components *k*_*x*_ and *k*_*z*_ across the interface, as a direct consequence of the phase matching of electromagnetic fields.

The tangential electric and magnetic field components are continuous at the interface:

49 $${C}_{1}{H}_{x,z}^{(1)}+{C}_{2}{H}_{x,z}^{(2)}={C}_{3}{H}_{x,z}^{(3)}+{C}_{4}{H}_{x,z}^{(4)},$$

50 $${C}_{1}{E}_{x,z}^{(1)}+{C}_{2}{E}_{x,z}^{(2)}={C}_{3}{E}_{x,z}^{(3)}+{C}_{4}{E}_{x,z}^{(4)},$$

where *C*_1_, *C*_2_, *C*_3_, and *C*_4_ are constants. The existence of a nontrivial solution of these constants requires that the determinant of the 4 × 4 matrix obtained from Eqs. (49) and (50) be zero, which gives the characteristic equation of the surface mode as

51 $$\begin{array}{c}{k}_{z}{k}_{0}^{2}\{{\alpha }_{+}{\alpha }_{-}({\alpha }_{-}{k}_{y}^{(3)}-{\alpha }_{+}{k}_{y}^{(4)})({k}_{y}^{(1)}{k}_{y}^{(2)}+{k}_{z}^{2})+{k}_{z}^{2}({\alpha }_{+}{k}_{y}^{(3)}-{\alpha }_{-}{k}_{y}^{(4)})\\ +\,{k}_{x}^{2}[{\alpha }_{-}({\alpha }_{+}^{2}-2){k}_{y}^{(4)}-{\alpha }_{+}({\alpha }_{-}^{2}-2){k}_{y}^{(3)}-4\varepsilon {\gamma }_{t}({k}_{y}^{(1)}+{k}_{y}^{(2)})]\}\\ -\,i{k}_{0}{k}_{x}{k}_{z}^{2}[2{\varepsilon }^{2}({k}_{y}^{(1)}{k}_{y}^{(2)}+{k}_{y}^{(3)}{k}_{y}^{(4)})+2({k}_{x}^{2}+{k}_{z}^{2})({\varepsilon }^{2}+{\gamma }_{t}^{2}-1)\\ -\,({k}_{y}^{(1)}+{k}_{y}^{(2)})({\alpha }_{+}{k}_{y}^{(3)}+{\alpha }_{-}{k}_{y}^{(4)})+2{\gamma }_{t}^{2}({k}_{y}^{(1)}{k}_{y}^{(2)}-{k}_{y}^{(3)}{k}_{y}^{(4)})]\\ +\,i{k}_{x}{k}_{0}^{3}\{{\alpha }_{+}{\alpha }_{-}^{2}[2{\alpha }_{+}({k}_{y}^{(1)}{k}_{y}^{(2)}+{k}_{z}^{2})-{k}_{y}^{(3)}({k}_{y}^{(1)}+{k}_{y}^{(2)})]\\ -\,2{k}_{z}^{2}({\varepsilon }^{2}+{\gamma }_{t}^{2})-{\alpha }_{+}{\alpha }_{-}{k}_{y}^{(4)}[{\alpha }_{+}({k}_{y}^{(1)}+{k}_{y}^{(2)})-2{k}_{y}^{(3)}]\}\\ +\,{\alpha }_{+}{\alpha }_{-}{k}_{z}{k}_{0}^{4}({\alpha }_{+}{k}_{y}^{(4)}-{\alpha }_{-}{k}_{y}^{(3)})+{k}_{z}^{3}{k}_{0}^{2}({\alpha }_{-}{k}_{y}^{(4)}-{\alpha }_{+}{k}_{y}^{(3)})=0.\end{array}$$

### Electromagnetic energy density

The time averaged energy density in a lossless bianisotropic medium is given by^46^

52 $$\langle W\rangle =1/4{V}^{\dagger }{M}_{0}V,$$

where

53 $${M}_{0}=(\begin{array}{cccccc}\frac{{\rm{\partial }}(\omega \varepsilon )}{{\rm{\partial }}\omega } & 0 & 0 & i\frac{{\rm{\partial }}(\omega {\kappa }_{t})}{{\rm{\partial }}\omega } & 0 & 0\\ 0 & \frac{{\rm{\partial }}(\omega \varepsilon )}{{\rm{\partial }}\omega } & 0 & 0 & i\frac{{\rm{\partial }}(\omega {\kappa }_{t})}{{\rm{\partial }}\omega } & 0\\ 0 & 0 & \frac{{\rm{\partial }}(\omega \varepsilon )}{{\rm{\partial }}\omega } & 0 & 0 & i\frac{{\rm{\partial }}(\omega {\kappa }_{z})}{{\rm{\partial }}\omega }\\ -i\frac{{\rm{\partial }}(\omega {\kappa }_{t})}{{\rm{\partial }}\omega } & 0 & 0 & \frac{{\rm{\partial }}(\omega {\mu }_{t})}{{\rm{\partial }}\omega } & 0 & 0\\ 0 & -i\frac{{\rm{\partial }}(\omega {\kappa }_{t})}{{\rm{\partial }}\omega } & 0 & 0 & \frac{{\rm{\partial }}(\omega {\mu }_{t})}{{\rm{\partial }}\omega } & 0\\ 0 & 0 & -i\frac{{\rm{\partial }}(\omega {\kappa }_{z})}{{\rm{\partial }}\omega } & 0 & 0 & \frac{{\rm{\partial }}(\omega {\mu }_{z})}{{\rm{\partial }}\omega }\end{array}),V=(\begin{array}{c}\sqrt{{\varepsilon }_{0}}{E}_{x}\\ \sqrt{{\varepsilon }_{0}}{E}_{y}\\ \sqrt{{\varepsilon }_{0}}{E}_{z}\\ \sqrt{{\mu }_{0}}{H}_{x}\\ \sqrt{{\mu }_{0}}{H}_{y}\\ \sqrt{{\mu }_{0}}{H}_{z}\end{array}),$$

with ${V}^{\dagger }$ being the Hermitian conjugate of *V*. The energy density must be positive definite, which implies that both the trace and determinant of *M*_0_ are positive:

54 $${\rm{Tr}}({M}_{0}) > \mathrm{0,}\,{\rm{Det}}({M}_{0}) > 0.$$

Based on the Lorentz model adopted in the present medium [cf. Sec. 3.3], these quantities become

55 $$\begin{array}{rcl}{\rm{Tr}}({M}_{0}) & = & \frac{3{\varepsilon }_{\infty }{\omega }^{4}-6{\varepsilon }_{\infty }{\omega }^{2}{\omega }_{0}^{2}+3{\varepsilon }_{\infty }{\omega }_{0}^{4}+2{\mu }_{t\infty }{({\omega }^{2}-{\omega }_{0}^{2})}^{2}+{\mu }_{z\infty }{({\omega }^{2}-{\omega }_{0}^{2})}^{2}}{{({\omega }^{2}-{\omega }_{0}^{2})}^{2}}\\  &  & +\,\frac{6{\omega }^{2}{\omega }_{0}^{2}{\Omega }_{\mu t}-2{\omega }^{4}{\Omega }_{\mu t}-{\omega }^{4}{\Omega }_{\mu z}+3{\omega }^{2}{\omega }_{0}^{2}{\Omega }_{\mu z}+3{\omega }_{p}^{2}({\omega }^{2}-{\omega }_{0}^{2})}{{({\omega }^{2}-{\omega }_{0}^{2})}^{2}}\end{array}$$

and

56 $$\begin{array}{ccc}{\rm{D}}{\rm{e}}{\rm{t}}({M}_{0}) & = & \frac{1}{{({\omega }^{2}-{\omega }_{0}^{2})}^{12}}\{-4{\omega }^{2}{\omega }_{p}^{2}{\omega }_{0}^{4}{\Omega }_{\gamma t}^{2}+[{\varepsilon }_{{\rm{\infty }}}{({\omega }^{2}-{\omega }_{0}^{2})}^{2}+{\omega }_{p}^{2}({\omega }^{2}+{\omega }_{0}^{2})]\\  &  & {[{\mu }_{t{\rm{\infty }}}{({\omega }^{2}-{\omega }_{0}^{2})}^{2}-{\omega }^{2}{\Omega }_{\mu t}({\omega }^{2}-3{\omega }_{0}^{2})]\}}^{2}\{-4{\omega }^{2}{\omega }_{p}^{2}{\omega }_{0}^{4}{\Omega }_{\gamma z}^{2}+\\  &  & [{\varepsilon }_{{\rm{\infty }}}{({\omega }^{2}-{\omega }_{0}^{2})}^{2}+{\omega }_{p}^{2}({\omega }^{2}+{\omega }_{0}^{2})][{\mu }_{z{\rm{\infty }}}{({\omega }^{2}-{\omega }_{0}^{2})}^{2}-{\omega }^{2}{\Omega }_{\mu z}({\omega }^{2}-3{\omega }_{0}^{2})]\}\end{array},$$

both of which are positive definite in the present study.

### Simulation

The simulation domain is on the *xy* plane and *k*_*z*_ is the out-of-plane wave vector component, which is kept fixed in the simulation so that the eigenwaves possess the same *k*_*z*_^24^. The surface mode is excited at a point on the boundary of the metamaterial, which can be implemented experimentally by a dipole antenna^14,18^. For the dipole to serve as the source of circular or elliptical polarization, the dipole has two in-plane components and the phase difference in between is set to be *π*/2 or −*π*/2 to mimic the right-handed or left-handed wave^50^. In experiment, the dipole source will excite the electromagnetic radiation for all wave numbers, and the measurement results are projected along the *k*_*z*_ direction. The surface modes with a particular value of *k*_*z*_ can be found in the contour plot of measurement^51^.
